# Disseminated gonococcal infection in a Japanese man with complement 7 deficiency with compound heterozygous variants: A case report

**DOI:** 10.1097/MD.0000000000025265

**Published:** 2021-04-02

**Authors:** Misaki Kageyama, Hideharu Hagiya, Yasutaka Ueda, Katsuki Ohtani, Yasuo Fukumori, Norimitsu Inoue, Nobutaka Wakamiya, Nanoka Yoneda, Keigo Kimura, Motonori Nagasawa, Futoshi Nakagami, Isao Nishi, Ken Sugimoto, Hiromi Rakugi

**Affiliations:** a Department of General Medicine; b Division of Infection Control and Prevention, Osaka University Hospital; c Department of General Medicine, Okayama University Graduate School of Medicine, Dentistry and Pharmaceutical Sciences, Okayama; d Department of Hematology and Oncology, Osaka University Hospital; e The Japanese Association for Complement Research; f Department of Clinical Nutrition, Rakuno Gakuen University; g Department of Molecular Genetics, Wakayama Medical University; h Laboratory section, The Japanese Association for Complement Research; i Department of Medicine and Physiology, Rakuno Gakuen University; j Laboratory for Clinical Investigation, Osaka University Hospital, Japan.

**Keywords:** complement addition test, complement deficiency, disseminated gonococcal infection, genome analysis, *Neisseria gonorrhoeae*, sexually transmitted infection

## Abstract

**Rationale:**

Complement deficiency are known to be predisposed to disseminated gonococcal infection (DGI). We herein present a case of DGI involving a Japanese man who latently had a complement 7 deficiency with compound heterozygous variants.

**Patient concerns:**

A previously healthy 51-year-old Japanese man complained of sudden-onset high fever. Physical examination revealed various skin lesions including red papules on his trunk and extremities, an impetigo-like pustule on left forearm, and tendinitis of his right forefinger.

**Diagnosis:**

Blood culture testing detected gram-negative cocci, which was confirmed to be *Neisseria gonorrhoeae* based on mass spectrometry and a pathogen-specific PCR test.

**Interventions:**

Screening tests for underlying immunocompromised factors uncovered that complement activities (CH50) was undetectable. With a suspicion of a congenital complement deficiency, genetic analysis revealed rare single nucleotide variants in complement 7 (C7), including c.281-1G>T and a novel variant c.1454C>T (p.A485V). CH50 was normally recovered by adding purified human C7 to the patient's serum, supporting that the patient has C7 deficiency with compound heterozygous variants.

**Outcomes:**

Under a diagnosis of DGI, the patient underwent an antibiotic treatment with cefotaxime for a week and was discharged without any sequela.

**Lessons:**

DGI is a rare sexually-transmitted infection that potentially induces systemic complications. Complement immunity usually defeats *N. gonorrhoeae* and prevents the organism from causing DGI. This case highlighted the importance of suspecting a complement deficiency when a person develops DGI.

## 1 Introduction

*Neisseria gonorrhoeae* infection is a very common sexually-transmitted disease with about 78 million cases per year worldwide,^[1]^ which usually causes localized genital infections in young adult populations. Infrequently (only about 0.5%–3% of the primary sexual mucosal infection),^[2]^ the pathogen invades into blood through the mucous membranes of the urethra, cervix, rectum, oropharynx, or conjunctivae,^[3]^ causing disseminated gonococcal infection (DGI). Patients with DGI manifest a wide variety of complications, such as tenosynovitis, dermatitis, and polyarthralgia, and even fatal infections including endocarditis, meningitis, and osteomyelitis.^[4]^ Recently, multidrug-resistant *N. gonorrhoeae* has globally emerged and the pathogen poses an emerging threat to the public health.^[5,6]^

Complement plays an essential portion of the innate immune system by purging bacteria from human body via pore-forming complexes.^[7]^ To date, an association of DGI and the complement deficiency (C1r,^[8]^ C2,^[9]^ C5,^[10]^ C6, C7,^[11,12]^ and C8^[12–14]^) has been reported, and we should note that patients with a complement deficiency possibly undergo refractory neisserial bacteremia.^[12]^ Even hypocomplementemia can undermine the prevention of gonococcal dissemination.^[8,15,16]^ As far as we concern, the literature has rarely described such a case in Japanese population. Herein, we describe a case of DGI that involved a Japanese man with complement 7 deficiency, which was confirmed by genome analysis and complement addition test.

## 2 Case presentation

**Figure 1:**
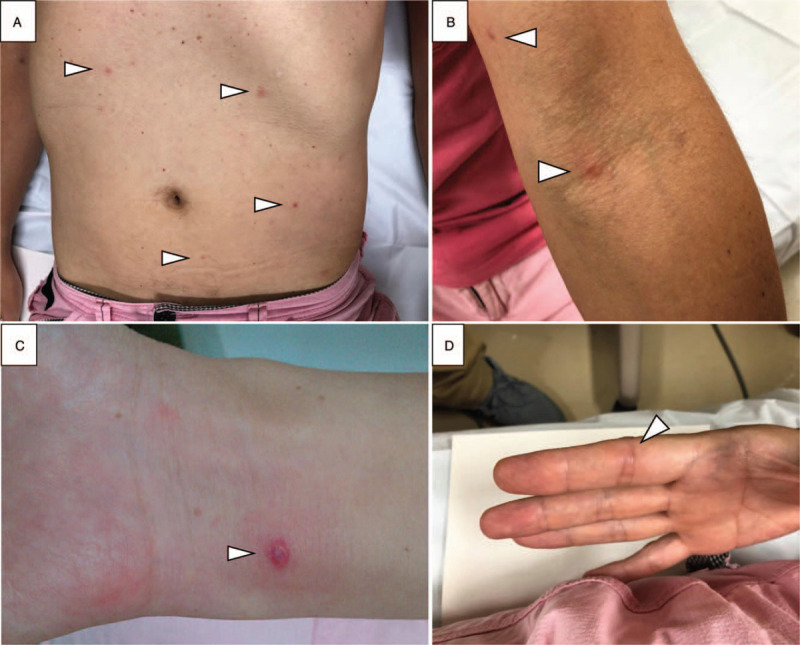
Skin manifestations of disseminated gonococcal infection. There were red papules on his trunk and extremities (A and B), an impetigo-like pustular lesion on left forearm (C), and tendinitis at proximal interphalangeal joint of right forefinger (D).

A 51-year-old man who experienced an acute-onset high fever presented to us. His past medical history included spontaneous pneumothorax, tonsillectomy, and Wolff–Parkinson–White syndrome. At the presentation, there was no respiratory, gastrointestinal, or urinary symptoms. He denied any previous history of repeated or refractory infectious episodes. His vital signs were as follows: body temperature, 38.8^o^C; blood pressure, 115/56 mm Hg; heart rate, 110 beats per minute, sinus, and oxygen saturation, 99% on room air. Physical examination revealed red papules on his trunk and extremities (Fig. 1A, B). A pustular lesion on right forearm showed an impetigo-like appearance (Fig. 1C), and the patient complained tendinitis at proximal interphalangeal joint of his right forefinger (Fig. 1D). Laboratory tests showed elevations of peripheral white blood cells (17,300 /μL), serum C-reactive protein (6.56 mg/dL), and procalcitonin (2.38 ng/mL); otherwise, there was no particular findings including urinalysis. Screening tests for sexually-transmitted infections such as syphilis, hepatitis B/C virus, and human immunodeficiency virus were all negative. Under a tentative diagnosis of a systemic febrile disease, and submitted blood culture samples (BD BACTEC FX blood culture system; Becton Dickinson, Sparks, MD). The patient was prescribed oral levofloxacin and returned home. Next day, gram-negative cocci was detected in the 2 sets of aerobic blood cultures, and the patient was hospitalized with suspicion of a systemic neisserial infection.

The pathogen could not be identified by matrix-assisted laser desorption/ionization-time of flight mass spectrometry (MALDI Biotyper; Bruker Daltonics, Bremen, Germany) directly using positive blood culture bottles. We subcultured the organism on 5% sheep blood (Becton Dickinson) and modified Thayer–Martin agar (Becton Dickinson). We then identified the pathogen as *N. gonorrhoeae* by means of colony-direct MALDI-TOF MS with a high score value of 2.405. A biochemical assay also confirmed it to be *N. gonorrhoeae* with an accuracy of >99% (ID test HN-20 rapid; Nissui, Tokyo, Japan). Further, we finally corroborated the organism to be *N. gonorrhoeae* via a specific PCR testing targeting the *piv* gene. The organism was not identified from intranasal, throat, and urine samples. On the basis of disk diffusion method, the organism was susceptible to cefotaxime and azithromycin, while resistant to ciprofloxacin and tetracycline.

After admission, we initiated cefotaxime (6 g per day). Fever as well as the various dermatologic manifestations subsided promptly, and the patient was discharged after 7 days of intravenous treatment. Later, detailed history taking revealed several episodes of sexual intercourse with commercial sex workers. The last sexual contact was 2 months before, and the patient denied any symptoms suggesting urethritis.

## 3 Investigations for complement deficiency

We suspected a presence of any underlying conditions, especially humoral-immune deficiency, for developing DGI in this patient. As a result of investigation, we found that C3 (120 mg/dL; reference range, 86–160 mg/dL) and C4 (33 mg/dL; reference range, 17–45 mg/dL) were within normal ranges, while complement activity (CH50) was undetectable. Considering a congenital complement deficiency, we submitted his blood sample to the Japanese Association for Complement Research with a consent from the patient. Whole exome analysis of 115 complement associated genes using MiSeq (Illumina, San Diego, California) found rare (allele frequency <0.005) heterozygous genetic variants in complement C7; c.281-1G>T at 3’ splice site of intron 4 and c.1454C>T (p.A485V) in exon 11. We confirmed that those variants are located on different chromosomes by sequencing amplified fragments from exon 5 to 7 including c.281-1G>T and fragments from exon 7 to 11 including c.1454C>T. CH50 of the patient's serum was recovered to normal range (49.0 CH50/mL) with a supplement of purified human C7 protein (50 μg/mL) in vitro, supporting that the patient has C7 deficiency with compound heterozygous variants.

## 4 Discussion

We presented a rare case of DGI that involved a patient with a complement 7 deficiency. Although similar cases have been reported worldwide,^[8–14]^ this may be the first such documented report in Japan accompanying a detailed genetic background. We could suspect the possibility of complement deficiency with the result of undetectable CH50 activity, despite normal activities of C3 and C4 components. The patient denied any previous history of repeated or refractory infections, possibly suggesting an immunocompromised condition. Thus, even the patient himself had never suspected the complement deficiency as his underlying disease until at his age.

A MEDLINE research uncovered that there have been 13 DGI cases in the literature in Japan^[16–18]^; however, an investigation for complement deficiency was not done in any of the cases. Homozygous but not heterozygous mutation c.281-1G>T has been previously reported to cause C7 deficiency.^[19]^ The alanine-485 is well preserved in vertebrate animals, suggesting that the novel variant c.1454C>T (p.A485V) is contributing to C7 deficiency in a compound-heterozygous manner in our case. CH50 recovery by adding purified C7 to the patient's serum supports this hypothesis.

Causal relationship between the C7 deficiency and development of DGI is yet to be elucidated in the literature. Terminal complement components, including C7, have been corroborated to be essential elements for preventing a development of invasive neisserial infections.^[20]^ Its complete deficiencies predispose patients to a great risk of recurrent neisserial infection, and in fact, patients with homozygous C7 deficiency are reported to be vulnerable to the severe disease during childhood and adolescence.^[21,22]^ Previously, it was estimated that individuals with C7 or C9 deficiency may develop invasive meningococcal disease at 10,000- or 1400-fold greater risk compared to those without the complement deficiency.^[23]^ Pathophysiologically, patients with compound-heterozygous terminal complement deficiency, like our patient, show undetectable CH50 activity as patients with homozygous deficiency does.^[23]^ Indeed, a molecular-based study described that 4 patients with C7 deficiency with compound heterozygous variants had undetectable CH50 activities, and had past histories of meningococcal systemic infections.^[24]^ Thus, we speculate that C7 deficiency with compound heterozygous variants would be a predisposing factor for DGI, as a consequence of zero-level CH50 activity. One previous review referred that lowered levels of terminal complement, which occurs as a result of heterozygous deficiency, can function to prevent the neisserial infection.^[21]^ In fact, among 13 young Japanese patients (19–42 years-old) with C5–8 deficiencies, 12 had no past medical history suggesting repeated infections.^[25]^

The terminal complement deficiencies are rare but should not be overlooked. According to a population-based screening test using blood donor samples collected at Osaka (Japan), the incidence of C9 deficiency was about 0.095%, accounting for most common complement deficiency in Japan.^[26]^ C7 deficiency is known to be the second most common type among complement deficiencies (0.0041%).^[25]^

DGI potentially yields various dermatologic presentations, such as hemorrhagic pustules, petechiae, and purpuric lesions.^[27]^ Our patient also manifested a variety of skin findings, including red papule, impetigo-like pustule, and tendinitis. In addition to a rarity of the disease, this diversity in presentation make it difficult to diagnose or suspect clinically. Physicians should remind of DGI in cases sexually active persons manifested pyrexia with various cutaneous findings.

A plausible reason for the prolonged latent period (2 months) between the last sexual contact and the onset of DGI was uncertain in this case. Though infrequent when compared to women, asymptomatic gonococcal infections are possible amongst men; latency periods of asymptomatic gonococcal infections could range from 3 to 154 days in men.^[28]^ Thus, we speculate that the pathogen could hide out somewhere in his body such as urethra and pharynx for that long term. Or, just simply, the patient might have withheld the truth about his real sexual contact history.

In conclusion, we reported a rare case of DGI in a Japanese man. Molecular exploration uncovered the patient to have C7 deficiency with compound heterozygous variants as an underlying disease. We should be aware that terminal complement deficiency, involving approximately 1 in 1000 Japanese people, potentially exposes a greater risk of severe neisserial infections.

## Author contributions

All authors meet the ICMJE authorship criteria: Misaki Kageyama, Hideharu Hagiya, Motonori Nagasawa, Futoshi Nakagami, Ken Sugimoto, and Hiromi Rakugi contributed to the patient management. Yasutaka Ueda, Katsuki Ohtani, Yasuo Fukumori, Norimitsu Inoue, and Nobutaka Wakamiya contributed to the analysis for complement deficiency. Nanoka Yoneda, Keigo Kimura, and Isao Nishi were responsible for bacterial identification. All authors contributed to the writing of the final manuscript.

**Data curation:** Isao Nishi.

**Formal analysis:** Nanoka Yoneda, Keigo Kimura, Isao Nishi.

**Methodology:** Nanoka Yoneda, Keigo Kimura, Isao Nishi.

**Supervision:** Hiromi Rakugi.

**Visualization:** Hideharu Hagiya.

**Writing – original draft:** Misaki Kageyama, Hideharu Hagiya, Yasutaka Ueda, Katsuki Ohtani, Yasuo Fukumori.

**Writing – review & editing:** Norimitsu Inoue, Nobutaka Wakamiya, Motonori Nagasawa, Futoshi Nakagami, Ken Sugimoto, Hiromi Rakugi.
